# Rapid single cell evaluation of human disease and disorder targets using REVEAL: SingleCell™

**DOI:** 10.1186/s12864-020-07300-8

**Published:** 2021-01-06

**Authors:** Namit Kumar, Ryan Golhar, Kriti Sen Sharma, James L. Holloway, Srikant Sarangi, Isaac Neuhaus, Alice M. Walsh, Zachary W. Pitluk

**Affiliations:** 1 Informatics & Predictive Sciences, Bristol Myers Squibb, Princeton, NJ 08648 USA; 2 Paradigm4, Inc., Suite 360, 281 Winter Street, Waltham, MA 02451 USA; 3 Informatics & Predictive Sciences, Bristol Myers Squibb, Redwood City, CA 94063 USA

**Keywords:** COVID-19, Coronavirus, ACE2, Data storage and retrieval, Information extraction, Virulence, Single cell analysis, SciDB, Array native database

## Abstract

**Background:**

Single-cell (sc) sequencing performs unbiased profiling of individual cells and enables evaluation of less prevalent cellular populations, often missed using bulk sequencing. However, the scale and the complexity of the sc datasets poses a great challenge in its utility and this problem is further exacerbated when working with larger datasets typically generated by consortium efforts. As the scale of single cell datasets continues to increase exponentially, there is an unmet technological need to develop database platforms that can evaluate key biological hypotheses by querying extensive single-cell datasets.

Large single-cell datasets like Human Cell Atlas and COVID-19 cell atlas (collection of annotated sc datasets from various human organs) are excellent resources for profiling target genes involved in human diseases and disorders ranging from oncology, auto-immunity, as well as infectious diseases like COVID-19 caused by SARS-CoV-2 virus. SARS-CoV-2 infections have led to a worldwide pandemic with massive loss of lives, infections exceeding 7 million cases. The virus uses ACE2 and TMPRSS2 as key viral entry associated proteins expressed in human cells for infections. Evaluating the expression profile of key genes in large single-cell datasets can facilitate testing for diagnostics, therapeutics, and vaccine targets, as the world struggles to cope with the on-going spread of COVID-19 infections.

**Main body:**

In this manuscript we describe REVEAL: SingleCell, which enables storage, retrieval, and rapid query of single-cell datasets inclusive of millions of cells. The array native database described here enables selecting and analyzing cells across multiple studies. Cells can be selected using individual metadata tags, more complex hierarchical ontology filtering, and gene expression threshold ranges, including co-expression of multiple genes. The tags on selected cells can be further evaluated for testing biological hypotheses. One such example includes identifying the most prevalent cell type annotation tag on returned cells.

We used REVEAL: SingleCell to evaluate the expression of key SARS-CoV-2 entry associated genes, and queried the current database (2.2 Million cells, 32 projects) to obtain the results in < 60 s. We highlighted cells expressing COVID-19 associated genes are expressed on multiple tissue types, thus in part explains the multi-organ involvement in infected patients observed worldwide during the on-going COVID-19 pandemic.

**Conclusion:**

In this paper, we introduce the REVEAL: SingleCell database that addresses immediate needs for SARS-CoV-2 research and has the potential to be used more broadly for many precision medicine applications. We used the REVEAL: SingleCell database as a reference to ask questions relevant to drug development and precision medicine regarding cell type and co-expression for genes that encode proteins necessary for SARS-CoV-2 to enter and reproduce in cells.

**Supplementary Information:**

The online version contains supplementary material available at 10.1186/s12864-020-07300-8.

## Background

Single cell RNA sequencing (scRNAseq) datasets have played a crucial role in identifying specific cell types in airway tissues that express the SARS-CoV-2 virus receptor, ACE2, and host responses in peripheral blood [1]. With more than 60 million cases of SARS-CoV-2 infection (COVID-19) and 1.4 million fatalities reported world-wide (26 November 2020) [2], SARS-CoV-2 interventions are an unmet medical need of pandemic proportions [3, 4]. Rapid identification of cell-type-specific expression and co-expression of the targets can identify novel cellular subtypes [5], facilitate decisions about biomarkers for target engagement [6] and response [7], potential delivery methods for therapies, and detection methods for diagnosis [8]. Additional host factors, TMPRSS2 and Cathepsin B/L, play a key role in the virus infection process and may be used as biomarkers and/or drug targets alone or in combination with ACE2. Peripheral responses may include the appearance of novel immune cellular subtypes and the absence of overexpression of traditional cytokine storm peptides [9]. COVID interactome map [10] serves as a rich resource set of approved medicines for testing once the tissue abundance is confirmed in COVID-19 patients.

While the field of precision medicine has steadily advanced through the elucidation of bulk tissue or fluid biomarkers, there is exciting potential for new discoveries due to scRNAseq. scRNAseq analysis is capable of identifying rare cell populations or markers on cellular subsets, associating cellular subsets with disease onset and/or treatment response. Single cell data collections like the COVID-19 Cell Atlas [11] (CCA) and the Human Cell Atlas [12] (HCA) are resources for expression profiling of key targets involved in SARS-CoV-2 infection of the cells and the subsequent immune response. However, the full utility of these data collections is limited due to a lack of database management strategy that allows facile cross comparison of the distribution and levels of specific gene expression between samples and projects without a significant bioinformatics and computational effort. For instance, determining the tissue distribution of expressed targets can enable rapid decisions for drug delivery methods and potential combination therapies. Without new data solutions, simple queries can become lengthy processes due to the scale of the datasets as well as the programming and computational resources required.

Ease of accessing and evaluating multiple scRNAseq data sets for the purposes of developing better therapeutic targets and biomarkers for clinical studies presents a fundamental challenge for their use in precision medicine. Seyhan et al. suggested that an important milestone for implementing precision medicine will be creating an “accessible data commons” to streamline biomarker discovery and simplify tests for the mechanism of action [13].. For the authors, the term accessible means easily searchable by non-programmer biomedical scientists for subsets of relevant data. The challenge is creating a data management and analysis capability that facilitates the comparison of small diseased tissue datasets, collected in the clinic, to other diseased tissue datasets in the public domain as well as to large healthy tissue datasets, like the Human Cell Atlas (HCA) [12]. These comparisons may identify the presence or emergence of subpopulations of cells that are resistant to therapy, or they could indicate infiltration or other cellular changes that would be elusive in either bulk RNAseq experiments or in flow cytometry, which are limited in the number of markers monitored [14]. The need for potentially high numbers of biological replicates to identify differential gene expression (DGE) will only accentuate the need for a data commons [15, 16].

This study describes the scalable REVEAL: SingleCell platform developed to address the issue of enabling rapid queries, simultaneously across multiple large single cell datasets stored in REVEAL: SingleCell, like the HCA, on the order of millions of cells. This study represents the first phase of a project to develop the framework necessary for searching across, analyzing, and in the future, implementing machine-learning in a data commons comprised of single cell precision medicine data sets. REVEAL: SingleCell addresses the challenge of storing large sparse arrays from various studies in a FAIR (findable, accesible, interoperable, reusable) manner. REVEAL: SingleCell is built on top of SciDB, an array native computational database that has R, Python, and REST APIs [17].

We loaded normalized scRNAseq data into the REVEAL: SingleCell platform to allow searching across reference datasets to find the distribution of transcripts for ACE2, TMPRSS2 and other host factors. The same schema and commands can be adapted for use with other single cell ‘omics data such as CITE-seq, snRNAseq and other data types. We provide timings for retrieving data that highlight the time challenges of the repetitive ETL (extract, transform and load) process that workflows like the Seurat [18] and HCAData [19] packages present.

### Construction and content

#### Construction

**Fig. 1:**
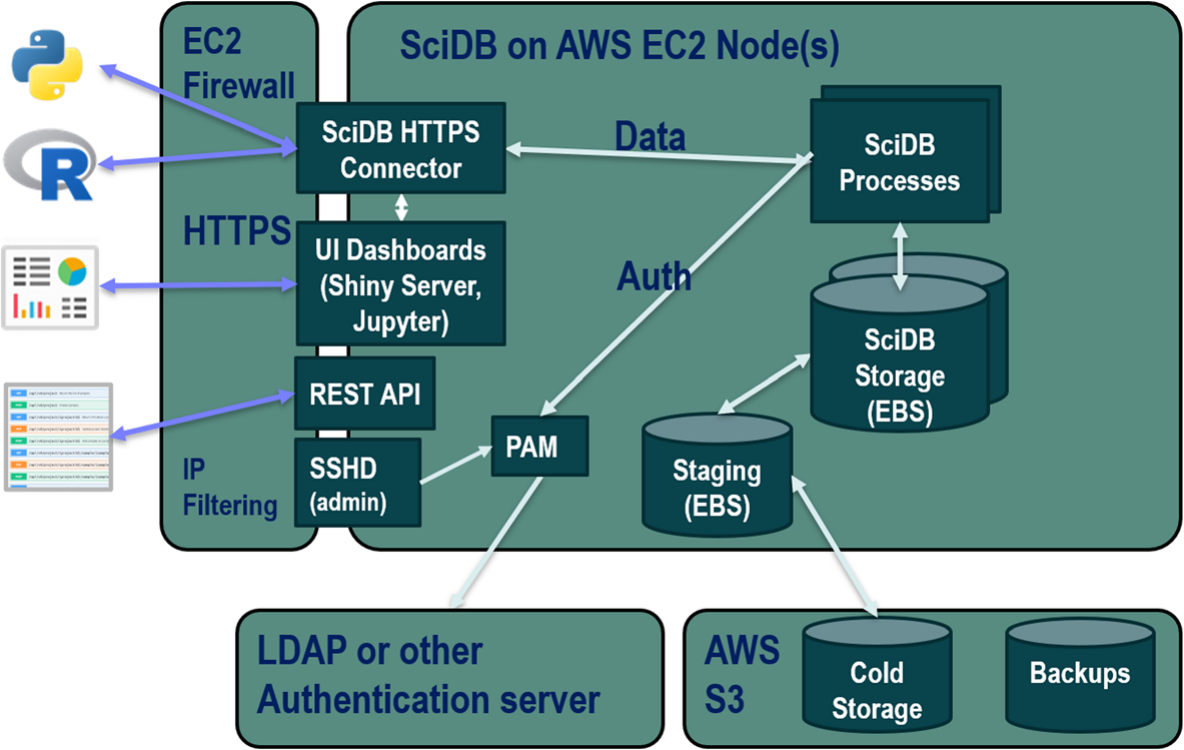
System configuration. REVEAL: SingleCell implementation in EC2. SciDB offers multiple paths to retrieve and load data. There are REST, R and Python APIs for server-side communication, R can also communicate via a local machine using HTTPS. The data and transactions are all ACID compliant. In this EC2 instance of REVEAL for scRNAseq, a 16-core machine with 64 GB of RAM, and 500 GB of SSD is used

Single cell data sets are loaded into SciDB, a unified scientific data management and computational platform organized around vectors and multi-dimensional arrays as the basic data modeling, storage, and computational unit [20]. The data model accommodates rapid and FAIR access to heterogeneous, multi-attribute data as well as metadata like ontologies and reference data sets. Multiple users can load, read, and write data in a secure, transactionally safe manner as data operations are guaranteed to be atomic and consistent (ACID compliant). The REVEAL: SingleCell solution is an app built on top of SciDB that provides purpose-built data schema, interfaces, and task-focused functionality, using controlled vocabulary. A Shiny GUI supports data visualization and exploration by non-programming scientists. R and Python APIs provide direct, ad hoc access and analysis, as well as extensibility via the integration of additional library packages. A FLASK [17] REST API implements a web interface. Documentation is provided as R markdown notebooks along with context-sensitive online help. Figure 1 provides a detailed view of the APIs, security, and storage architecture for SciDB implemented on AWS.

The software versions used are shown below in Table 1.

**Table 1:** Software requirements

Software	Version	URL
Linux	CentOS 7.5 / Ubuntu 14.04	
SciDB	19.11.5	
R	3.6	https://www.r-project.org
R packages		
- Seurat	3.1.x	https://satijalab.org/seurat/
- SciDBR	2.0.2	github.com/Paradigm4/scidbr
- revealgenomics	0.6	private github
- revealsc	0.1.0	private github
Python	3.7.6	https://www.python.org
Python REST API	1.1.2	https://flask.palletsprojects.com/en/1.1.x/

Legend: the list of software versions used for analysis

#### Content

The following publicly available datasets were loaded: Human Cell Atlas (HCA) Census of Immune Cells data set [21], COVID-19 Cell Atlas (CCA) [11] (excluding the Aldinger, et al. Fetal Cerebellum data set). These datasets were all aligned to the GRCh38 reference genome. Data sizes into the hundreds of TBs are feasible. The current system contains 32 projects, totalling less than 1 TB.

HCA provided filtered raw counts data in 10x CellRanger version 3.0 format. This data was loaded into R as a Seurat object, normalized using the Seurat scTransform algorithm [22] and then converted back to 10x CellRanger v3.0 format. The CCA provided normalized data in .h5ad format as used in the Python Scanpy [23] and anndata [24] libraries. CCA .h5ad files were converted into the 10x CellRanger format (using standard convertors from the Python anndata, scipy.io [25] libraries). In both cases, the cell metadata tags (e.g., CellType, percent.mt) were saved as .tsv files from the normalized Seurat object (HCA) and .h5ad files (CCA), and loaded into the database using the REST API metadata update endpoints. The REST API checked for consistency in the 10x format, missing values, among others.

#### Content schema

Data are modelled as multi-dimensional arrays on disc. Each element in an array contains one or more attributes. Storing the data on disc as arrays (or vectors) enables rapid sub-setting of cells by gene expression levels, ontology and QC tags, individually and in combination across samples.

**Fig. 2:**
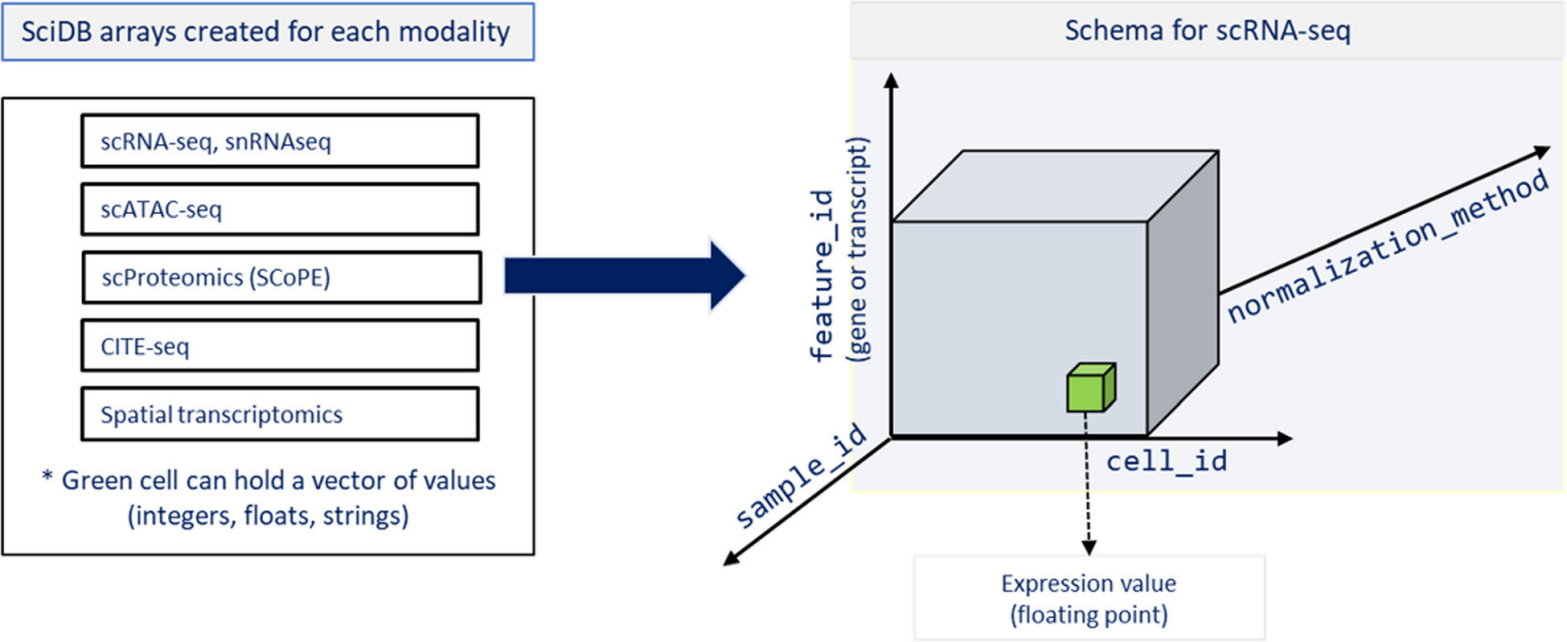
Single cell data types compatible with REVEAL: SingleCell

Figure 2 illustrates the various single cell data submodalities that can also be stored in the array elements of the n-dimensional SciDB arrays. Although this project stored only scRNAseq data, the multi-dimensional array schema can be extended to hold many complimentary data types, including snRNAseq, scATAC-seq, CITE-seq, among others.

Elements in the n-dimensional arrays can contain several orthogonal omics data types, as mentioned in the figure.

#### Content data hierarchy

**Fig. 3:**
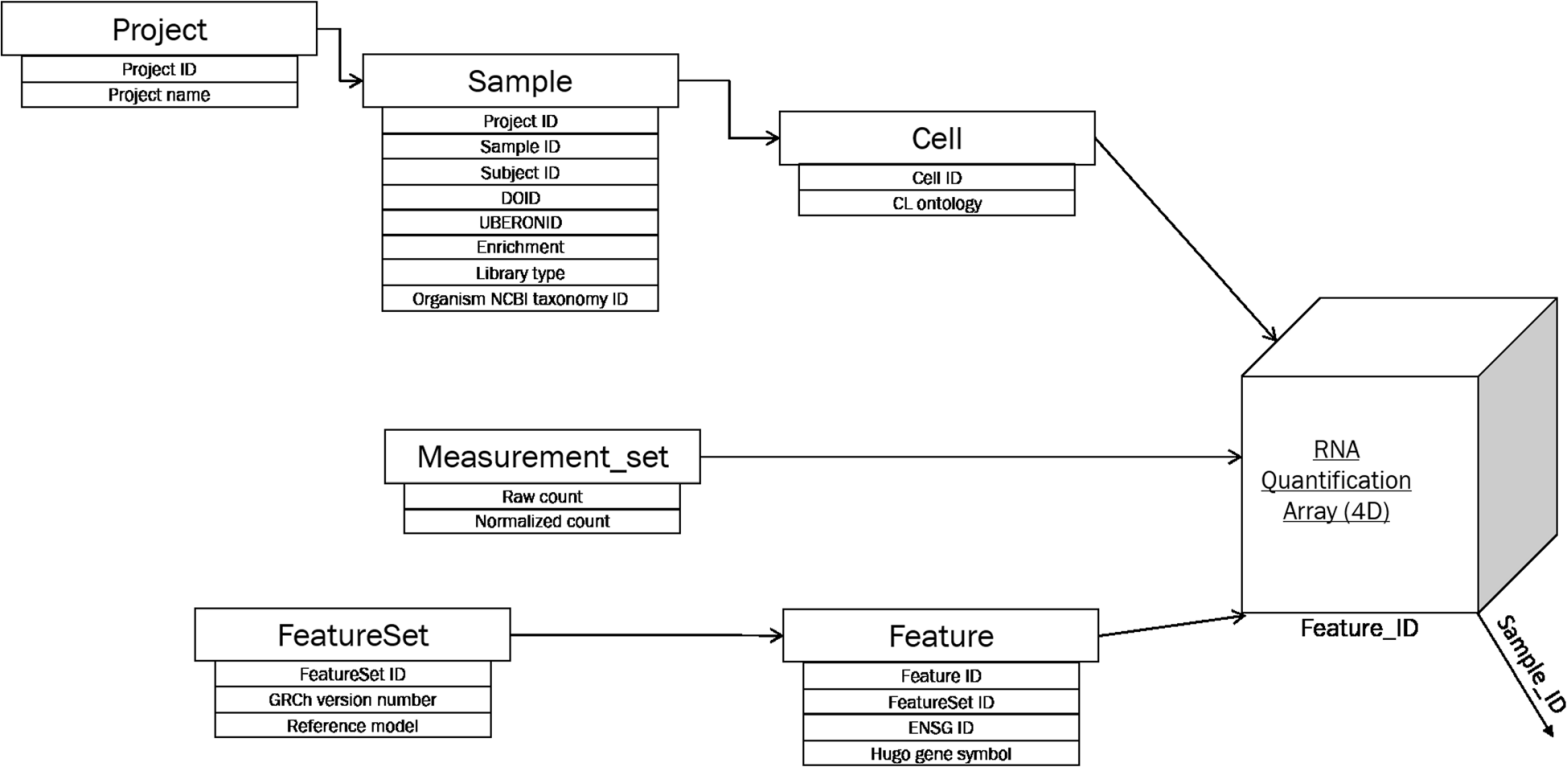
Meta data hierarchy of REVEAL: Single Cell

Figure 3 illustrates the hierarchical relationship of metadata. The label “projects” was used for collections of samples which are often also referred to as studies. For instance, the HCA Census of Immune Cells is one project with 16 samples. At the sample level, anatomy/tissue type and disease type are selectable as filters with the UBERON and DOID identifiers. At the cell level, CL IDs were used to enable selection of specific cell types. It is important to note that there was tremendous heterogeneity in how the metadata was presented in these individual projects on the atlas website, and an automated system for unification is being developed. Feature sets [26] include information about the human genome version and the sub-category feature, allowing selection by either ENSEMBL ID or gene symbol. Gene symbols were used because most public data are not annotated with ENSEMBL IDs. Due to the diversity of the metadata (especially when sourced from public studies), we stored metadata as key-value pairs in the elements of the sample array shown below in Table 2.

**Table 2:** Arrays and attributes in REVEAL: SingleCell

Array	Dimensions	Attribute data types	Attributes
RNAQUANTIFICATION	sample_id^1^ measurementset_id^1^ cell_id^1^ feature_id^1^	value: float	Raw count, normalized count
SAMPLE	sample_id^2^	name: string description: string project_id: int64^1^ public: bool	Project ID, Sample ID, Subject ID, DOID, UBERONID, Enrichment, Library type, Organism NCBI Taxonomy ID Assay type
MEASUREMENTSET describes how the data was collected and processed.	measurementset_id^2^ sample_id^1^	experimentset_id: int64^1^ entity: string name: string description: string featureset_id: int64^1^	
CELL	cell_id^2^ sample_id^1^	name: string description: string individual_id: int64^1^	CL ID, Cl ontology
FEATURE (Genes) Features can also be proteins, other biomolecules, and or hierarchical names.	featureset_id^1^ gene_symbol_id^1^ feature_id^2^	name: string gene_symbol: string chromosome: string start: string end: string feature_type: string source: string	Feature ID, Featureset ID, ENSG ID, Hugo gene symbol
FEATURE SET	featureset_id^2^		GRCh version, Reference model, Feature-set ID
PROJECT FEATURE describes the project, or datasource like HCA	project_id^2^	name: string description: string project_id: int64^1^	Project name, Project ID

Legend: shows the schema. Data of interest can be accessed and filtered by their dimensions and attributes. The superscript 1 indicates primary dimensions for selection, and the superscript 2 inidcates secondary dimensions for selection. The general categories for attributes include but are not limited to:

▪ scRNAseq expression values, both normalized and raw counts

▪ categorical and continuous tags which can contain metadata on any entities from the pipeline used to generate the tags.

- projects, e.g. data generation source (public, institutional -internal)

- samples, e.g. UBERONID; DOID; organ (lung, rectum, illium)

- cells, e.g. CL ID; cell type (CD8+, enterocytes); percent.mt (percent mitochondria)

- features, e.g. strand (+, −); biotype (protein-coding, frameshift)

Assay type (10x or Dropseq, …)

Note that the tags, UBERONID, DOID, and CL ID, hold controlled vocabulary from publicly curated ontologies like Ontobee. These tags enable hierarchical searches, e.g. search for all cells matching CLID CL:0000584 (enterocyte) and its children

#### Content data curation

Cell type is one of the most important selection criteria. However public datasets in CCA used multiple disparate naming conventions, e.g. cell_type, CellType, celltypes, celltype1. These names were retained as is in the database, but an extra tag, CellType.select, was added for harmonization across all projects. The CellType.select tag was manually curated.

Subject-level and sample-level metadata were often missing in the CCA. We provide a manually curated supplementary table with the exact numbers of subjects and samples, where it was possible to obtain the information (S1).

#### Queries and REST API

Table 3 lists the queries and functions built into the REVEAL: SingleCell app.

**Table 3:** Queries and functions built into the database

HCA whitepaper requirements	Functionality	Functionality in Reveal: SingleCell
At least one developer-oriented portal providing a platform (e.g. FireCloud or Toil) in which developers can bring containerized environments to perform analyses on the data		R & Python API allow users to work in R studio or Python and directly select data from REVEAL: SingleCell
At least one user-oriented portal providing interactive interfaces to the data; for example:		R & Python API
	Quantifying the expression of a given gene (e.g., marker genes specified by user) across cell types, shown in several popular modalities (e.g., low-d plots, heatmaps, violin plots)	SingleCellviewer and Plotly connecting to REVEAL: Singlecell R & Python API
	Showing clustering of individual cells from an experiment based on expression profiles;	R & Python API clustering routines
	Painting cell clusters (ordinations) by metadata (technical and experimental) to identify batch effects and visualize biological groupings (depending on the type of metadata);	SingleCellviewer and Plotly connecting to REVEAL: Singlecell R & Python API
	Visualizing gene signatures by several modalities, including heatmaps and dot plots of average expression by cell group; and	SingleCellviewer and Plotly connecting to REVEAL: Singlecell R & Python API
	Cross-correlating gene expression with epigenetic markers.	Using the REVEAL: SingleCell R & Python API
Multiple query-oriented portals with APIs targeting custom access patterns, for example: Tag based queries		
	Querying all gene expression tables generated with a particular analysis	Using the REVEAL: SingleCell Rest API & R notebook
	Querying all cells for those that match the expression pattern of a target cell and return the metadata for the matching cells	Using the REVEAL: SingleCell Rest API & R notebook
	Querying all raw data for a specific tissue type, ranked based on a custom combination of quality-control metrics.	Using the REVEAL: SingleCell Rest API & R notebook
Housekeeping requirements		
	Loading data	Using the REVEAL: SingleCell Rest API & R notebook
	Adding tags after data load	Using the REVEAL: SingleCell Rest API & R notebook
	Deleting data	Using the REVEAL: SingleCell Rest API & R notebook

Legend: The requirements listed in the HCA whitepaper take two forms: actual queries and visualization capabilities. The R and Python APIs support the visualization requirements. The REST API and R notebook support the queries. We included the housekeeping requirements in the list because those are essential capabilities for a database

**Fig. 4:**
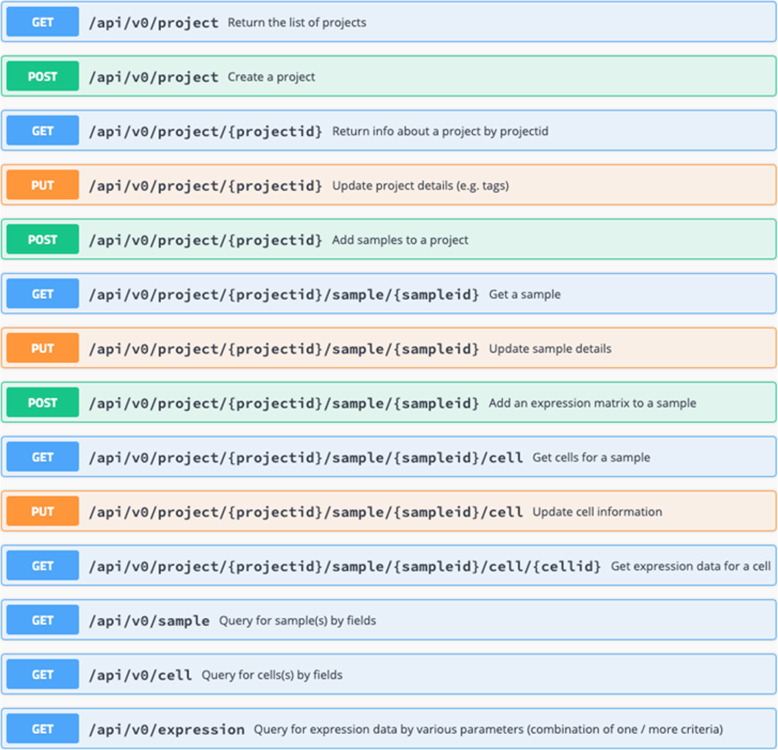
REST API Fig. 4 shows the different GET, PUT and POST commands needed to query, upload and update data. Data is loaded from custom scRNAseq processing pipeline where data is output in the 10x Genomics Cell Ranger format and read in using the Seurat library READ10X function. The file names are barcodes.tsv, features.tsv, and matrix.mtx

These are accessible through an R API and REST API. Figure 4 lists the REST API commands.

## Utility and discussion

We approached the challenges of creating a data commons by deploying a scientific computational database, SciDB. There are distinct benefits to having scRNAseq data organized as arrays in SciDB, such as allowing cross-study selection of cells by gene expression thresholds or metadata tags and analysis by multiple users, while ensuring the consistency from a shared version of QC’d data and workflows. SciDB endows REVEAL: SingleCell with future-readiness, the capability of integrating genomic, proteomic, image and metabolomic data types into the same database, enabling a data commons.

Many researchers use Seurat objects or HDF5 files for storage of both scRNAseq data and calculated results. This approach contradicts the basic concept of FAIR data because each object is a silo of data. Cross-study analysis with Seurat requires loading the studies of interest into a single Seurat object and repeating a Seurat object merge step for each desired set of studies and is often limited by RAM**.** Thus, analysis is limited by the size of the compute hardware, i.e. RAM, to fewer than 1 million cells. Yet, the outlook is for dataset sizes to grow especially when coupled with flow cytometry, microscopy and new methods. For example, single cell and single nucleus data sets range in complexity from analysis of total mRNAs, to capped RNAs to transcriptional velocity to transient physiologic responses [27], many of which may be inter-compared to test hypotheses [28]. Emerging higher throughput and lower cost methods of single cell transcriptional profiles like Sci-Plex, will create much larger data sets to search across and analyze [29].

REVEAL: SingleCell was designed as a data commons with the goal of removing silos, supporting cross-study analysis, and enabling scaling of computation beyond a single instance. We populated the REVEAL: SingleCell platform with scRNAseq data from the HCA and CCA (content and construction). The same schema and commands can be used with other single cell ‘omics data such as CITE-seq [30] and snRNAseq data [31].

As a design guide, we implemented the requirements for querying data outlined in the HCA whitepaper. The HCA whitepaper didn’t include provisions for an actual database; storage was based on file retrieval. The requirements for precision medicine put a premium on being able to inter-compare datasets without needing increasingly larger amounts of RAM.

- Querying all gene expression data generated with a particular analysis,
- Querying all cells for those that match the expression pattern of a target cell and return the metadata for the matching cells; and
- Querying all raw data for a specific tissue type, ranked based on a custom combination of quality-control metrics.

Table 2 shows the schema, a collection of 7 arrays. This schema fulfills the requirements for queries laid out in Table 3, allowing sub-setting of cells by gene expression levels, ontology and QC tags, individually and in combination across samples. Using the REVEAL: SingleCell platform, more complex queries relating to ontologies as well as to gene expression levels (or other continuous variables like x, y coordinates or time), or patterns can be combined. This is enabled because each element in an n-dimensional SciDB array can have unlimited numbers of tags that can be used for selection (Table 2, Fig. 3). Thus, users can:

- Query for gene expression in cells matching a cell type, and then expand the search to include cell types that are parents or children in a cell type ontology.
- Query for gene expression to return cells with gene expression above, below, or within thresholds (e.g., ACE2 > 3, < 7, 4–6).
- Query raw and/or normalized counts for each cell.

### Applying REVEAL: SingleCell to evaluate key regulators involved in SARS-CoV-2 infection

**Fig. 5:**
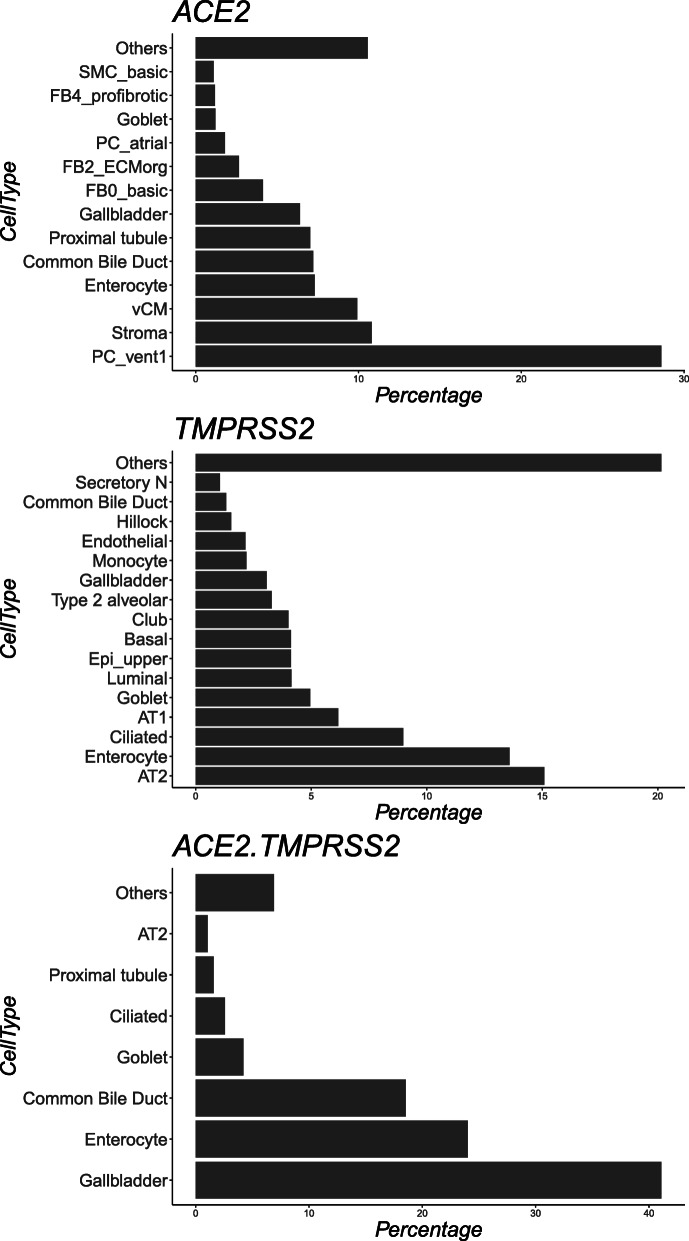
Shows a bar graph of the results from table 5, cell type occurrence across the datasets in REVEAL: SingleCell. Panel A shows the ranking of cell type occurrence for cells expressing ACE2. Panel B shows the ranking of cell type occurrence for cells expressing TMPRSS2. Panel C shows the ranking of cell type occurrence for cells expressing both ACE2 and TMPRSS2

In this early phase of SARS-CoV-2 research, hypotheses regarding tissue/cell type distribution of host cofactors for viral infection (receptors, processing enzymes) and pathogenesis (changes in normal cell gene expression profiles) need to be tested quickly. As an illustration of the capabilities of REVEAL: SingleCell, we queried for all cells in the database (datasets from CCA, HCA) that either express the receptor for SARS-CoV-2, *ACE2*, the cell surface receptor for SARS-CoV-2 [32], and entry facilitating enzyme, transmemembrane serine protease, *TMPRSS2* [33], or co-express both mRNAs with *DPP4*, the receptor for MERS-CoV [34] (Tables 4 and 5, and Fig. 5). An example of a more complex query is shown (Table 4, query 6): sequentially applying a metadata filter and then a gene expression filter on the results. These findings highlight that REVEAL: SingleCell returned results that can support interactive hypothesis generation and testing by searching across more than 30 datasets in a timespan of seconds.

**Table 4:** Benchmarking queries

Capabilities	Search criteria	Query #	Search result # of cells returned (# of projects with data)	Total time (sec)
**Selecting a subset of cells (searching across 2.2 M cells and 32 projects)**				
By tags	1 tagCellType.select is any of: [‘Enterocyte’, ‘Enterocytes’, ‘Best4+ Enterocytes’, ‘Enterocyte Progenitors’, ‘Immature Enterocytes 1’, ‘Immature Enterocytes 2’]	1	19 K cells (5 projects)	14
	2 tags Above criteria on CellType.select & Location is any of: [‘Rectum’, ‘Decidua’, ‘Ileum’]	2	4827 cells (2 projects)	9
**Selecting cells across projects**				
Checking for co expression in more than 1 gene, when expression value lies in a range • Threshold: value > = 1 • Restricting search to normalized data	‘ACE2’, ‘TMPRSS2’	3	2282 cells (21 projects)	26
	‘ACE2’, ‘TMPRSS2’, ‘DPP4’	4	561 cells (11 projects)	32
**Search expression**				
By gene list across all projects	‘ACE2’, ‘TMPRSS2’, ‘DPP4’	5	225 K rows (32 projects; download size: 8 MB)	15
By cells across multiple projects	Using the result of Query 1 to search expression on those cells i.e. searching by ~ 19,000 cells (in the projects with data)	6	26.7 M rows (5 projects; download size: 1019 MB)	27
By selected project	Project: ‘wang20_rectum’; matrix_count: ‘normalized’	7	11.6 M rows (1 project; download size: 621 MB)	17

Legend: Queries were organized as: searching by metadata tags (1 & 2), searching by co-expression (3, 4), searching by gene list (5), searching the results of query 1 by expression levels (6), and returning the results of a project

**Table 5:** Cells matching search criteria are grouped by their cell type tags and the table reports percentages of cell type tags in total cells matching search criteria. Cell types with < 1% prevalence of total cells matching search criteria, are grouped together as “Others”

Percentage Pos cell - ACE2		Percentage Pos cell - TMPRSS2		Percentage Pos cell - Co-expression - ‘ACE2’, ‘TMPRSS2’	
PC_vent1	28.61	AT2	15.09	Gallbladder	41.1
Stroma	10.82	Enterocyte	13.59	Enterocyte	24.01
vCM	9.93	Ciliated	8.98	Common Bile Duct	18.54
Enterocyte	7.31	AT1	6.17	Goblet	4.21
Common Bile Duct	7.22	Goblet	4.96	Ciliated	2.59
Proximal tubule	7.04	Luminal	4.15	Proximal tubule	1.58
Gallbladder	6.41	Epi_upper	4.13	AT2	1.05
FB0_basic	4.13	Basal	4.12	Others	6.92
FB2_ECMorg	2.66	Club	4.02		
PC_atrial	1.8	Type 2 alveolar	3.29		
Goblet	1.22	Gallbladder	3.08		
FB4_profibrotic	1.18	Monocyte	2.2		
SMC_basic	1.1	Endothelial	2.16		
Others	10.57	Hillock	1.54		
		Common Bile Duct	1.32		
		Secretory N	1.05		
		Others	20.16		

Legend: Cells matching search criteria are grouped by their cell type tags (Fig. 5) and the table reports percentages of cell type tags in total cells matching search criteria. Cell types with < 1% prevalence of total cells matching search criteria, are grouped together as “Others”

Table 4 lists the times to return an R data frame in RStudio from querying REVEAL: SingleCell for the listed queries across many or all of the samples from CCA and HCA.

We evaluated multiple samples from CCA and HCA to identify cell type tag of cells expressing *ACE2*, *TMPRSS2*, and co-expression of both the markers. All cells matching the above criteria were grouped together by their cell type tags and reported as percentage of total cells matching criteria. Cell type tags with < 1% of total cells matching criteria were grouped together and labelled as ‘Others’ and the sum of their percentanges was also reported.

Our analysis, based on all cells currently loaded in the database (Fig. 5), highlights that the majority of cells expressing *ACE2* have a cell type tag of PC_vent1 (Heart tissue); the majority of cells expressing *TMPRSS2* have a cell type tag of AT2 (alveolar epithelial type II cells found in the lung parenchyma); and most cells co-expressing both *ACE2* and *TMPRSS2* are tagged as Gall bladder cells. These results are consistent with previous studies [1, 35–37].

These results, in part, explain the the multi-organ involvement in infected patients observed worldwide during the on-going COVID-19 pandemic, as multiple cell types in the human body express genes utilized by SARS-CoV-2 for infection. REVEAL: SingleCell enables quick profiling of key genes involved in the current pandemic and supports additional use cases that require evaluation across a large database of single cell expression datasets such as vaccine candidates for infectious diseases, biomarkers for oncology patient stratification, and immunology-related disorders.

## Conclusion

In this paper, we introduce the REVEAL: SingleCell database that addresses immediate needs for SARS-CoV-2 research and has the potential to be used more broadly for many precision medicine applications. We used the REVEAL: SingleCell database as a reference to ask questions relevant to drug development and precision medicine regarding cell type and co-expression for genes that encode proteins necessary for SARS-CoV-2 to enter and reproduce in cells.

### Significance

The COVID-19 atlas used in this project is an example of an extensive reference dataset that can be used for understanding individual patient responses to novel therapies relative to untreated and un-infected patient data. Implementation of REVEAL: SingleCell harnesses the power of working with large and complex single cell datasets and unlocks their potential by significantly speeding up the process of selecting and analyzing data for understanding and treating individual patients using precision medicine. The next phase of development will be to extend the REVEAL: SingleCell architecture to include additional relevant datasets, as well as include other omics data types from single cell experiments.

## Supplementary Information

**Additional file 1.**

## Data Availability

Data is available at these websites: COVID-19 Cell Atlas. https://www.covid19cellatlas.org. Accessed 06 June 2020. Human Cell Atlas Data Portal: Mapping the human body at the cellular level. https://data.humancellatlas.org. Accessed 06 June 2020.
