# Supplementary material for: Rapid single cell evaluation of human disease and disorder targets using REVEAL: SingleCell™
Source: BMC Genomics. 2021 Jan 6;22:5. doi: 10.1186/s12864-020-07300-8 (PMC7785925; doi:10.1186/s12864-020-07300-8)
Supplement: Supplementary file 1 — Additional file 1. [file 12864_2020_7300_MOESM1_ESM.pdf]

Table S1

Covid-19 Cell Atlas Metadata Validation:

The Covid-19 Cell Atlas (www.covid19cellatlas.org) at the time of publication consists of 35 projects (datasets), out of which 34 projects were utilized for this paper. Aldinger et al. was not used due to inconsistencies in the matrix file.

The process of compiling the metadata (e.g., number of unique donors, number of samples) started by exploring the *'cells.tsv'* file, which was available for each project. We found that the fields that could identify the metadata in the *'cells.tsv'* files were not standardized among the different projects. There were projects with different fields for the same attribute (e.g., 'donor' or 'Source' for unique donors), or no relevant field at all.

We then proceeded to go through each project's associated publication, GEO accession entries (if any), and supplementary data files. Values obtained from this exploratory search for fields such as unique number of cell-types, donors, samples were corroborated by analyzing the *'cells.tsv'* file as well as the *'.mtx'* file for each project. For projects that have unpublished results, we contacted the researchers to confirm metadata information.

Project Name	Number of Unique Biological Samples (Donors)	Number of Cells	Comments	Assay Type: Library prep, sequencing instrument, alignment
aldinger_cerebellum_restricted.processed	NA	71553	Unpublished work	Illumina TruSeq RNA Access Prep Kit, Illumina HiSeq 2000, STAR
cheng18.processed	Uncertain	68036		10x Chromium 3' v2, (Illumina HiSeq 2500, HiSeq 4000, NovaSeq 6000), STAR
habib17.processed	Uncertain	13067		Illumina Nextera, Illumina NextSeq 500, STAR
henry18_0.processed	Uncertain	32921	Text in paper suggests only 3 donors out of a total 11 used for sc analysis; GEO entry also suggests 3 donors; cell numbers in dataset do not match	10x Chromium 3' v2, Illumina NextSeq 500, STAR
lako_cornea.processed	NA	21343	Unpublished work	NA
miller20.processed	Uncertain (8 anatomical samples)	36807	Samples identified by 'Sample' in cells.tsv	10x Chromium, Illumina HiSeq 4000, STAR
popescu19.processed	Uncertain (14 donors likely - see Comments)	95461	cells.tsv field 'batch' can be parsed to obtain just the suffix which shows 14 donors. The EBI metadata file shows 14 donors but the id's do not match	(10x Chromium 3' v2 and V(D)J, Illumina Nextera XT (for SS2)), Illumina HiSeq 4000, STAR

stewart19_adult.processed	Uncertain (Potentially 2 donors - see Comments)	40268	From HCA: Study has 8 donors in total - 6 fetal and 2 mature. This dataset seems to have the 2 mature donors based on reported cell count in HCA and the publication. Pre-print version mentions different number of donors	10x Chromium v2, NA, STAR
vallier_restricted.processed	NA	8414	Unpublished work	NA, NA, NA
vento18_10x.processed	Uncertain (7 donors most likely - see Comments)	59705	Project 'Source' (EBI) on covid19cellatlas.org suggests 6 donors, and the cells.tsv is missing a few replicates for 3 of the 6 donors; On HCA, appears like there are 11 donors (for 10x); Publication states 7 donors for 10x	10x Chromium v2, Illumina HiSeq 4000, STAR
vento18_ss2.processed	Uncertain (5 donors most likely - see Comments)	4891	Project 'Source' (EBI) does not have metadata info for the SS2 sequenced samples (at least the link from covid19cellatlas.org does not); Paper states 5 donors for SS2	Smart-seq2, Illumina HiSeq 2000, HISAT2
deprez19_restricted.processed	10 Donors	77969		10x Chromium 3' v2, Illumina NextSeq 500/550, STAR
baron16.processed	4 donors	7714	batch' field in cells.tsv has 12 unique specimens. The suffix 'human1_' is an identifier for the 4 human donors (3 replicates each for donor 1 & 2; donor 3 has 4 replicates;	Illumina CEL-SEQ, Illumina HiSeq 2500, STAR

			donor 4 has 2 replicates)	
guo18_donor.processed	3 donors, 6 samples (2 replicates each)	6199	Each replicate for a donor has a suffix 'Donor_1_rep1' and 'Donor_1_rep2' - this leads two 6 unique 'Source' entries (but only 3 unique donors)	10x Chromium 3' v2, Illumina HiSeq 2500, STAR
james20.processed	5 donors	41650	number of cell types for each donor (21-25)	10x Chromium 3' v2 and 5' v2, (Illumina HiSeq 4000 and Illumina NovaSeq 6000), STAR
litvinukova20_restricted.processed	14 donors (6 different regions sampled from each donor)	467939		10x Chromium, NA, STAR
lukassen20_airway_orig.processed	4 donors	17451		10x Chromium 3' v2, Illumina HiSeq4000, STAR
lukassen20_lung_orig.processed	12 donors	39778		10x Chromium v2, Illumina HiSeq 4000, STAR
macparland18.processed	5 donors	8444		10x Chromium 3' v2, Illumina HiSeq 2500, STAR
madissoon20_oesophagus.processed	6 donors; 23 specimens	87947		10x Chromium 3' v2, Illumina HiSeq 4000, STAR
madissoon20_spleen.processed	5 donors; 19 specimens	94257		10x Chromium 3' v2, Illumina HiSeq 4000, STAR
martin19.processed	11 donors	32458	Only 'Uninvolved' ileal samples in data set	10x Chromium 3' v2, Illumina NextSeq 500, STAR
menon19.processed	3 donors; 6 specimens	20091		10x Chromium 3' v3, Illumina HiSeq 4000, STAR
park20.processed	23 donors; 74 specimens	255901	Project 'Source' (EBI) on covid19cellatlas.org shows 24 donors (cells.tsv has 23 - missing donor is A45 with only 1 sample)	(10x Chromium 3' and 5', Illumina Nextera (for SS2)), Illumina HiSeq 4000, STAR
smillie19_epi.processed	12 donors	34772	Study has 30 donors (18 with Ulcerative Colitis and 12 Healthy controls); this dataset only contains the 12 healthy donors (id's match between	(10x Chromium 3' v2 and v3; GemCode v1), (Illumina NextSeq and HiSeq), STAR

			cells.tsv and supplementary metadata from publication)	
vieira19_Alveoli_and_parenchyma_anonymised.processed	6 donors	12971		10x Chromium 3', Illumina HiSeq 4000, STAR
vieira19_Bronchi_anonymised.processed	7 donors (most likely)	16873	cells.tsv shows 7 unique donor id's. The supplementary data spreadsheet also suggests 7 donors. Publication shows 9 donors (broken up into 2 groups, so there might be an overlap). Could not confirm donor id's with another source	10x Chromium 3', Illumina HiSeq 4000, STAR
vieira19_Nasal_anonymised.processed	2 donors	7087	cells.tsv shows 2 unique donor id's. The supplementary data spreadsheet also suggests 2 donors. Publication shows 2 donors.	10x Chromium 3', Illumina HiSeq 4000, STAR
voigt19.processed	3 donors; 6 specimens	4335	Each donor has 2 types of specimens (macula and peripheral) - 6 in total. (Study looks at 7 donors, but donors 4-7 are in expt 2, not in this dataset)	10x Chromium v3, Illumina HiSeq 4000, STAR
wang20_colon.processed	2 Donors	4329		10x Chromium 3', Illumina HiSeq X Ten PE150, STAR
wang20_ileum.processed	2 Donors	5980		10x Chromium 3', Illumina HiSeq X Ten PE150, STAR
wang20_rectum.processed	2 Donors	3797		10x Chromium 3', Illumina HiSeq X Ten PE150, STAR
